# Quaternary Interaction of the HIV-1 Envelope Trimer with CD4 and Neutralizing Antibodies

**DOI:** 10.3390/v13071405

**Published:** 2021-07-20

**Authors:** Qingbo Liu, Peng Zhang, Paolo Lusso

**Affiliations:** Laboratory of Immunoregulation, National Institute of Allergy and Infectious Diseases, NIH, Bethesda, MD 20852, USA; peng.zhang2@nih.gov (P.Z.); plusso@niaid.nih.gov (P.L.)

**Keywords:** HIV-1, CD4, receptor binding, viral entry, quaternary interaction, neutralizing antibodies

## Abstract

The entry of HIV-1 into host cells is initiated by the interaction of the viral envelope (Env) spike with the CD4 receptor. During this process, the spike undergoes a series of conformational changes that eventually lead to the exposure of the fusion peptide located at the N-terminus of the transmembrane glycoprotein, gp41. Recent structural and functional studies have provided important insights into the interaction of Env with CD4 at various stages. However, a fine elucidation of the earliest events of CD4 contact and its immediate effect on the Env conformation remains a challenge for investigation. Here, we summarize the discovery of the quaternary nature of the CD4-binding site in the HIV-1 Env and the role of quaternary contact in the functional interaction with the CD4 receptor. We propose two models for this initial contact based on the current knowledge and discuss how a better understanding of the quaternary interaction may lead to improved immunogens and antibodies targeting the CD4-binding site.

## 1. Introduction

The envelope (Env) spike is a key structural and functional component of HIV-1 because it mediates viral attachment and entry into target cells and, therefore, it is the sole target of virus-neutralizing antibodies [1,2]. The Env spike is a trimeric glycoprotein comprised of three identical gp120-gp41 heterodimers. Through interaction with the CD4 receptor expressed on target cells, the Env undergoes a series of dramatic conformational changes that lead to the exposure or formation of the binding site for the coreceptors CCR5 or CXCR4. After gp120 binds to the coreceptor, the CD4–gp120 complex dissociates from gp41, which contains the fusion peptide at its N-terminus, and the membrane fusion process is initiated [1,2]. Because of its metastable nature and trimeric composition, the HIV-1 Env has been a difficult target to investigate. Recent advancements in structural biology have dramatically improved our knowledge of the Env trimer structure, particularly after its stabilization by mutation or interaction with different ligands [3,4,5,6,7,8,9,10,11,12,13,14]. This review is focused on our current understanding of the initial binding of the Env trimer to the CD4 receptor, which was recently shown to involve a quaternary interaction with two contiguous gp120 protomers. The functional consequences of this initial quaternary contact and the implications for the design of new inhibitors and immunogens will also be discussed.

## 2. Primary CD4-Binding Site

The CD4 glycoprotein, which is primarily expressed on the surface of CD4^+^ T cells and monocyte/macrophages, was identified as the main cellular receptor for HIV-1 soon after the discovery of the virus [15,16]. The CD4-binding site (CD4-BS) in the gp120 Env subunit was initially investigated by mutagenesis [17,18,19,20,21]. In 1998, the first structure of gp120 complexed with a soluble form of CD4 (sCD4) and an antibody to a CD4-induced (CD4i) epitope, 17b, was solved, providing the first high-resolution information on gp120 and atomic details of its interaction with CD4 [22]. CD4 was shown to bind to gp120 through its D1 domain, the first of its four immunoglobulin-like extracellular domains (D1–D4). This primary CD4-BS is comprised of multiple discrete regions mainly from the gp120 outer domain. Although the Env sequence is highly variable, the key residues that make direct contact with CD4 are relatively conserved, as is the interaction mode across various divergent isolates [22,23,24,25]. However, in all the early reports, CD4 was complexed with monomeric gp120, which adopts a post-fusion structure that does not accurately reflect the conformation of the membrane-anchored pre-fusion trimeric spike. A first attempt to characterize the trimeric state was made by Liu and colleagues, who reported 3D reconstructions of native Env trimers on virion particles by electron microscopy (EM) at ~20 Å resolution [26]. By fitting crystal structures of gp120 into the maps of unliganded, b12-bound or CD4/17b-bound trimers, they proposed a model for the Env conformational changes that occur as a consequence of receptor interaction. The unliganded native timer was shown to adopt a closed conformation. Upon CD4 binding, however, the Env trimer becomes fully open, with the three gp120 protomers rotating outward and the D1D2 domains of CD4 bending toward the host cell surface to bring the virus closer to the cellular membrane [26]. However, the low resolution of these structures did not provide any further insights into the gp120 interface with CD4 or its intramolecular conformational changes. In 2013, the generation of soluble, truncated and stabilized HIV-1 Env trimers such as the BG505 SOSIP trimer [27] provided a long-awaited tool for studying the structure of the trimeric Env. These trimers adopt a near-native antigenic conformation, as shown by their recognition by the majority of broadly neutralizing antibodies (bNAbs) and their limited interaction with non-neutralizing antibodies [27]. In addition, they maintain functional competence, as CD4 binding induces conformational changes that result in the exposure of CD4i epitopes. A series of high-resolution X-ray and cryo-EM structures have henceforth been reported, illustrating the atomic details of the prefusion configuration of the HIV-1 Env spike in most studies complexed with various neutralizing antibodies, which contributed to stabilizing the trimeric structure [3,4,5,8,11,28,29,30,31,32,33]. Moreover, a few studies have investigated the structure of the open or partially open trimer in complex with soluble CD4 and/or anti-CD4i antibodies [34,35,36,37,38,39]. These studies confirmed the composition and structure of the primary CD4-binding site, as previously defined using monomeric gp120.

## 3. Quaternary CD4-Binding Site

**Figure 1:**
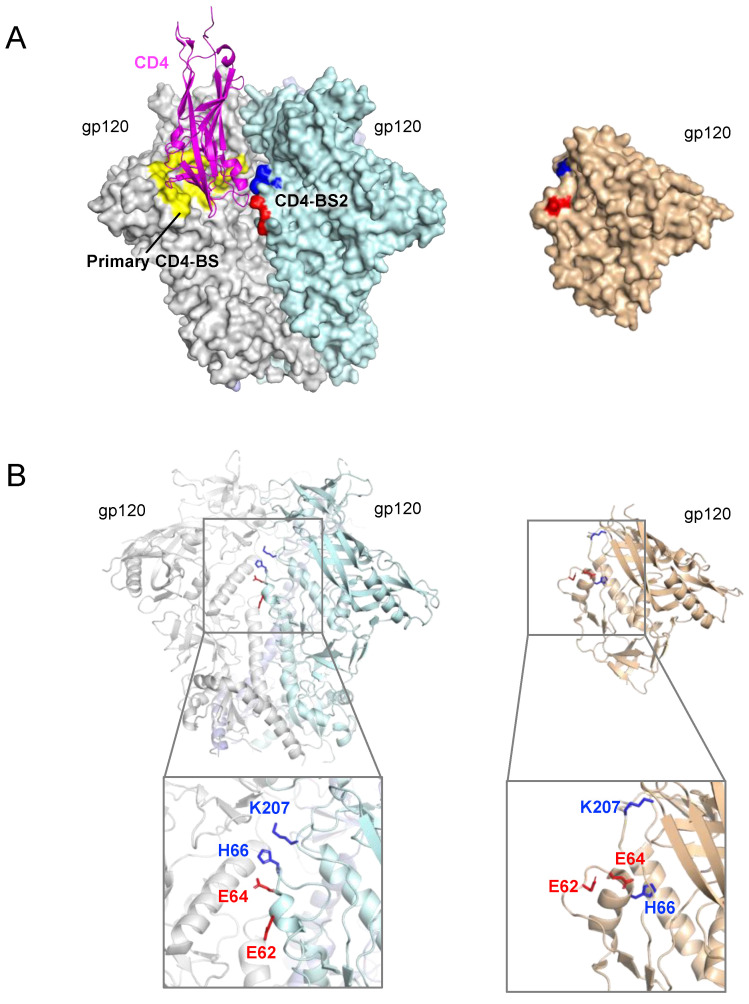
Structure of the CD4-BS2 in a SOSIP.664 Env trimer (PDB ID: 4TVP) and in a gp120 monomer (PDB ID: 4OLU). (**A**) Left, primary CD4-BS (yellow) and CD4-BS2 (red and blue) are shown on the trimeric Env. The CD4 molecule is docked onto the trimer by aligning its complexed gp120 moiety to one gp120 protomer of the Env trimer. Right, the amino acid residues that form CD4-BS2 are highlighted in the monomeric gp120 structure. (**B**) Residues of CD4-BS2 are shown as sticks and colored by their charges (blu = positive; red = negative). As shown, the side chains of key CD4-BS2 residues adopt opposite orientations in the two conformations of the gp120 subunit: outward and fully solvated in the trimer (**left**); inwards and predominantly solvent inaccessible in the monomer (**right**).

Despite some lingering controversy [40,41,42,43,44], SOSIP trimers are generally accepted in the field as close proxies of the native Env form and, thereby, have offered an opportunity to explore the critical initial events in the interaction between the Env spike and the CD4 receptor. In 2017, using a combination of in silico docking, functional mutagenesis and cryo-EM analysis, we reported that this initial interaction has a quaternary nature, as it involves not only the primary CD4-BS, but also a previously unrecognized second CD4-BS (that we defined CD4-BS2) located in the outer layer (layer-1) of the inner domain of a neighboring gp120 protomer. We started by performing in silico modeling of the molecular complex between CD4 and the prefusion trimer in an attempt to project the initial interaction before the induction of conformational changes. Thus, we aligned the published structure of a CD4-bound gp120 monomer to one gp120 protomer of a SOSIP trimer. The primary CD4-BS on the trimer aligned well with that of monomeric gp120. However, CD4 seemed to reach deep into the interprotomer groove of the trimeric structure to establish additional contacts with a second gp120 protomer (Figure 1A). A potential quaternary contact of CD4, albeit less extensive, was also postulated by other authors based on structural modeling, but not experimentally investigated [4]. We identified four highly conserved amino acids in the inner domain of gp120, namely, E62, E64 and H66 in the α$\bar{1}$-helix, and K207 at the base of the β3–β4 loop, as potential sites of direct contact with CD4 (Figure 1B). The α$\bar{1}$-helix of gp120 is a flexible region, as indicated by its poor resolution in several SOSIP trimer structures or by the variable side chain orientation of its key residues [5,11,12,14,30,45,46], with the four predicted contact residues being projected outwards in the trimeric structure but flipping inwards to contact layer 2 of the inner domain in monomeric gp120 (Figure 1B). To explore the functional role of CD4-BS2, we introduced both alanine and charge-inversion mutations in this region and showed that they caused not only a significant decrease in CD4-binding affinity but also a dramatic reduction in infectivity in different HIV-1 strains and clades. Interestingly, mutations in CD4-BS2 impaired the ability of the Env trimer bind to CD4 by dramatically reducing the off-rate of the interaction, while the on-rate was only minimally altered [9]. These results support a key role of CD4-BS2 in the functional interaction of the Env spike with the CD4 receptor. An alternative explanation for the loss of CD4 binding and infectivity induced by mutations in CD4-BS2 could be a putative allosteric effect caused by disruption of the two-layer arrangement of the gp120 inner domain [47]. However, such interpretation is implausible because it is based on results obtained with monomeric gp120, which has a completely different structure in this region, and because the charge-inversion mutations that caused the most dramatic loss of function in the complete trimer only altered the electrostatic charge of fully solvated amino acid side chains, which point outwards and have no contacts whatsoever with the underlying layer 2 of the gp120 inner domain.

**Figure 2:**
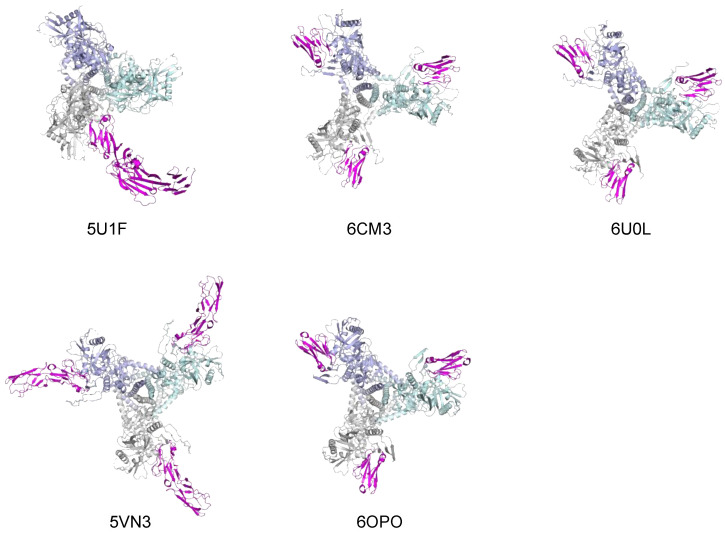
Structures of CD4 complexed with SOSIP.664 trimers in closed (5U1F), partially open or open states (6CM3, 6U0L, 5VN3, 6OPO). CD4 is colored in magenta. The three gp120 protomers of the Env trimers are colored in gray, cyan and blue, respectively. Other stabilizing ligands in each structure were removed for clarity.

As mentioned above, the engagement of CD4 causes the Env trimer to open, starting with the centrifugal translation of the three gp120 protomers, which progressively diverge and, eventually, fall off the gp41 trimer base. This implies that the quaternary contact between CD4 with two neighboring gp120 protomers can only represent a transient intermediate step in the chain of conformational changes initiated by CD4 binding. Indeed, as stated above, the solvent-exposed, CD4-accessible orientation adopted by CD4-BS2 in the prefusion trimer no longer exists in the low-energy post-fusion gp120 structure. Thus, it is reasonable to assume that the quaternary contact is one of the earliest events in Env–CD4 interaction. Whether this quaternary contact precedes, occurs simultaneously with, or follows the primary CD4 contact remains to be elucidated. To visualize this initial contact, a conformationally constrained soluble trimer, DS-SOSIP.664, was utilized for structural studies. The DS-SOSIP.664 binds to CD4 asymmetrically with a 1:1 stoichiometry and remains locked in the prefusion conformation even after CD4 binding [48]. The cryo-EM structure of the DS-SOSIP.664 complexed with 4-domain sCD4 and a trimer-specific antibody, PGT145, was solved [9]. The structure confirmed that the trimer maintains its closed state after CD4 binding (Figure 2, 5U1F). Besides the classic interaction with the primary CD4-BS of one gp120 protomer, this structure provided direct experimental evidence that CD4 establishes quaternary contact with CD4-BS2, as predicted by our in silico model. This quaternary contact is lost in partially or fully opened trimers complexed with CD4 (Figure 2, 6CM3, 6U0L, 5VN3 and 6OPO). CD4-BS2 appears to contribute ~23% of the total interface of CD4 binding. Interestingly, a ~3 Å outward shift of the two CD4-interactive gp120 protomers toward the incoming CD4 molecule was observed, which results in a slight increase in the width of the other two interprotomer grooves. This shift may represent the earliest conformational change induced by CD4 binding in the Env trimer. The initial trimer asymmetry induced by CD4, which is consistent with previous observations by single-molecule FRET analysis [49], suggests that the first receptor contact is mediated by a single CD4 molecule, which in turn facilitates the addition of other CD4 receptors to the complex. Of note, the relatively low resolution of the cryo-EM structure (6.8 Å) did not allow for a fine definition of the quaternary contacts. However, a higher-resolution structure has recently been obtained and is currently being refined for publication (Priyamvada Acharya, personal communication). Preliminary results are consistent with the previously defined structure of the CD4-BS2 region.

**Open questions:**

- What is the temporal sequence of contacts that occur during HIV-1 Env–CD4 interaction: does the primary or the quaternary interaction occur first? Or do they occur simultaneously?
- What is the stoichiometry of the initial Env-CD4 contact? Is binding of a single CD4 molecule to the Env trimer a mandatory initial step before other CD4 molecules can be added to the complex?
- What is the evolutionary purpose for HIV-1 to adopt a quaternary receptor contact?

## 4. Proposed Models of the Quaternary Env-CD4 Contact

**Figure 3:**
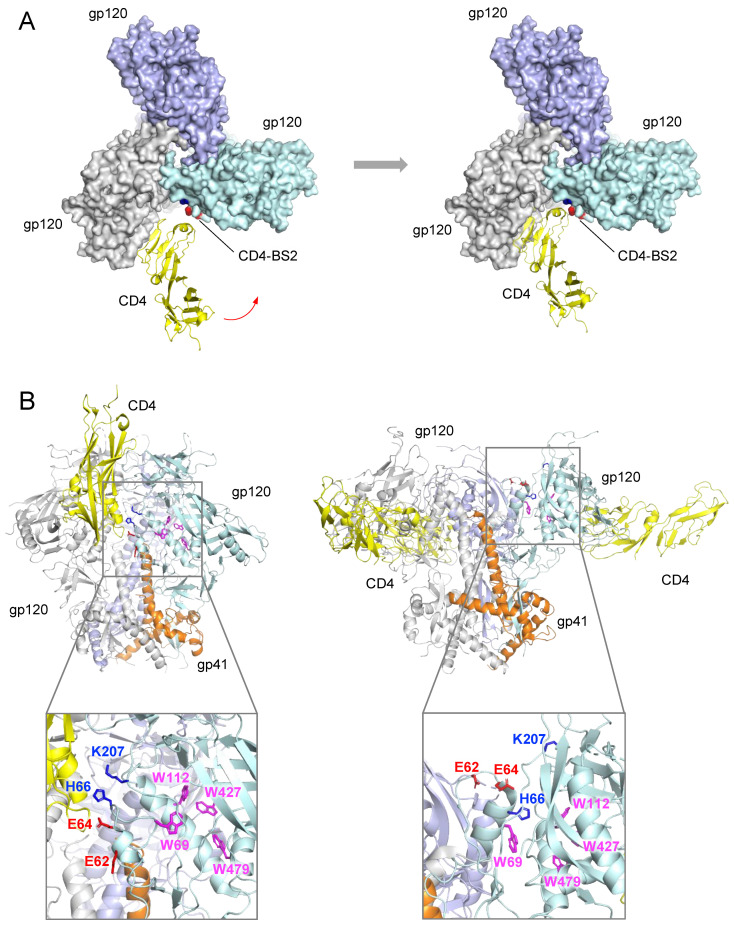
Models of CD4-BS2 function in the HIV-1 entry process. (**A**) Hold-and-position model. Left panel: top view of a predicted unstable state with a single CD4 molecule (taken from structure 3JWD) bound only to the primary CD4-BS of the HIV-1 Env trimer (PDB ID: 4TVP). The red arrow denotes the direction of CD4 movement towards the neighboring gp120 protomer. Right panel: top view of the CD4 molecule bound with an optimal angle that engages both the primary CD4-BS and CD4-BS2. Residues on the the surface of CD4-BS2 are colored in blue and red. (**B**) Touch-and-go switch model: side view of a closed Env trimer (PDB ID: 4TVP) and a partially open trimer (PDB ID: 5VN3). Left panel: a monomeric gp120 structure complexed with CD4 is aligned to one protomer of the SOSIP Env trimer, showing the close contact of CD4 (yellow) with the CD4-BS2 (positively charged E62 and E64 in red, negatively charged H66 and K207 in blue). The tryptophan cavity is colored in magenta and gp41 in orange. Right panel: a partially open trimer complexed with CD4 and antibody 17b (removed for clarity). The residues are color coded as in the left panel.

The identification of CD4-BS2 provided new insights into the initial contact of CD4 with the HIV-1 Env trimer. However, many questions are still largely unresolved. In particular, it is still unclear how CD4-BS2 functions in facilitating the process of viral entry, and it is challenging to create a coherent model of the molecular events that follow the initial CD4 binding. Nevertheless, there is a series of important experimental clues that may help to understand the role of CD4-BS2:

1. Single-molecule FRET analysis has provided evidence that the interaction of CD4 with the Env trimer is a sequential multi-step process that proceeds through discrete conformational states starting from the prefusion unliganded state (referred to as state-1) and ending with the final 3:1 CD4-bound state (state-3) [50]. Of note, these conformational changes transition through an asymmetrical intermediate (state 2), in which a single CD4 molecule is bound to the Env trimer [49]. This intermediate state seems to correspond to the single CD4-bound trimer structure [9], which also shows an asymmetry, with a ~3 Å outward shift of the two CD4-interactive gp120 protomers toward the incoming CD4 molecule. In this structure, however, further downstream conformational changes were hampered by the covalent disulfide bond between gp120 positions 201 and 433 engineered into the DS-SOSIP trimer. These findings corroborate the hypothesis that the earliest Env–CD4 contact is established with a single CD4 molecule.
2. A CD4-mimetic peptide, M48U1, is able to induce the complete set of CD4-like conformational changes without making contact with CD4-BS2, raising questions about the effective requirement for the secondary binding site for triggering trimer activation [39,51,52]. However, M48U1 is a small molecule, which can easily reach the primary CD4-BS without facing steric hindrances from the adjacent gp120 protomer or surrounding glycans. Thus, this observation suggests that CD4-BS2 acts as a facilitator that helps CD4 overcome the steric hurdles that limit its access to the closed Env trimer. However, this explanation is difficult to reconcile with the fact that mutations in CD4-BS2 dramatically reduce the off-rate of CD4 interaction, with only a limited impact on the on-rate [9]. This observation suggests that contact with the primary CD4-BS is the main determinant of the on-rate, while CD4-BS2 acts as a stabilizer after the first contact is established.
3. As stated above, the α$\bar{1}$-helix, which encompasses three of the four main residues that form CD4-BS2, is a very flexible region, as shown by its poor resolution in several SOSIP structures. This property underlies the ability of this region to rapidly dissipate by adopting a radically different conformation and releasing CD4 during the transition toward the open trimer conformation. Likewise, its structural rearrangement may also release critical intramolecular interactions with other gp120 and gp41 domains that play a role in maintaining the Env in its high-energy metastable prefusion state.
4. The unique position of CD4-BS2, adjacent to the N-terminus of the gp41 α7-helix and directly overlapping a large tryptophan cavity that is critical for CD4 interaction, suggests that CD4-BS2 may play a mechanistic role in the induction of the conformational changes that lead to the opening of the Env trimer (Figure 3). One of the four tryptophan residues that form the cavity is actually within the α$\bar{1}$-helix and snaps out of the cavity in CD4-bound gp120, with its position being occupied by H66. Thus, it is possible that the binding of CD4 to CD4-BS2 may play a role in perturbing the interactions of the inner domain layer 1 with the gp41 bundle and the tryptophan cavity, releasing key local constraints and thereby triggering conformational changes in these regions.

Based on the above observations, we propose two possible models to interpret the functional role of CD4-BS2 in the HIV-1 entry process: (i) the hold-and-position model (Figure 3A); and (ii) the touch-and-go switch model (Figure 3B). According to the hold-and-position model, although the primary CD4-BS contributes the majority of the CD4 binding interface, this contact faces steric hindrance from the neighboring protomer and surrounding glycans, which poses significant energy barriers and restricts the angle of approach of CD4. Once CD4-BS2 is engaged, it increases the binding energy, positions CD4 in the right orientation for an optimal interaction with the closed trimer and allows enough contact time for CD4 to trigger subsequent and irreversible conformational changes (Figure 3A). If only a single CD4 molecule initially engages the trimer, as we postulate, the early conformational changes in the two contact gp120 protomers seem to exert allosteric effects on other regions, creating an asymmetry that facilitates the subsequent engagement of two additional CD4 molecules. Once the conformational transition of the Env has started, the quaternary contact between CD4 and CD4-BS2 is disrupted by the divergence of the three gp120 protomers and by local conformational alterations. The entire process is mainly driven by the primary CD4-BS within the primary gp120 contact protomer, which has full competence for triggering the CD4-induced conformational changes in Env. 

An alternative model is the touch-and-go switch model, whereby CD4-BS2 plays a more substantive role in triggering conformational changes in the Env trimer. According to this model, the CD4-BS2 region is a key regulator of the closed trimer conformation that contributes to maintaining the underlying tryptophan cavity fully occupied and the gp41 α7-helix adherent to gp120. Binding of CD4 to CD4-BS2 induces local structural rearrangements that release these constraints and allow F43 of CD4 to gain full access into the tryptophan cavity owing to the partial snap-out of W69, while concomitantly freeing the gp41 α7-helix from its gp120-bound position (Figure 3B). Both of these events initiate irreversible downstream conformational alterations, including the inward folding and disappearance of CD4-BS2 itself, which, therefore, rapidly detaches from CD4. This transient touch-and-go interaction is consistent with the inherent flexibility of the CD4-BS2 region. 

Although the two models differ, they are not mutually exclusive because CD4-BS2 might, in fact, both serve to stabilize/position CD4 for an optimal interaction with the primary CD4-BS and, at the same time, participate in the triggering of downstream conformational changes by acting as a transient switch, along with the primary CD4-BS, in a tightly coordinated series of structural rearrangements. To explore the fine details of the initial contact and its immediate downstream effects, high-resolution structures of CD4 in complex with the native, closed Env trimer and its earliest intermediate states are needed. In this respect, solving the structure of CD4-BS2 mutants frozen in an intermediate conformational state may provide important information on the early structural changes induced by CD4 binding.

**Open questions:**

- Does the quaternary contact with CD4-BS2 only facilitate/stabilize the Env–receptor interaction or is it also critical for the induction of downstream conformation changes?
- How can a mini-CD4 molecule trigger CD4-like conformational changes without establishing contact with CD4-BS2?
- Can the immune system produce antibodies that selectively target the flexible CD4-BS2?

## 5. Stabilizing CD4-BS2 for Functional Studies and Immunogen Design

Because of its metastable nature and rapid conformational transitions upon CD4 binding, the HIV-1 Env is a difficult target for immunogen design. Due to the overall failure of conventional vaccine approaches using gp120 or near-native stabilized (SOSIP) trimers as immunogens, diverse strategies for stabilizing the Env trimer have been developed [10,53,54,55]. The main goal of these efforts was to prevent the opening of the trimer and fix it in its prefusion, closed state. Since CD4-BS2 is involved in the early events of CD4 binding and has a dramatically altered conformation in CD4-bound gp120, it could be explored as a target region for trimer stabilization. Mutations in CD4-BS2 were found not only to impair CD4 binding, but also to stabilize the trimer in a state that has a reduced binding to the CD4i mAb 17b and virtually no binding to mAb 48d and CCR5 [9]. Mutation of E64 or H66 together with other modifications were used to improve the properties of the soluble SOSIP.664 trimer. The resultant constructs showed reduced V3 accessibility and CD4-induced conformational changes [53]. The E64K and H66R substitutions were shown to occur spontaneously in escape variants after treatment with a peptidic anchor inhibitor, VIR165 [56]. The escape viruses were resistant to CD4 triggering but were able to infect target cells in the presence of VIR165 [56]. These studies suggest that altering the CD4-BS2 region may be an effective means to stabilize the Env trimer and might be helpful for HIV-1 immunogen design. Alternative strategies for stabilizing this region could be to introduce specific disulfide bonds or to engraft the α$\bar{1}$-helix onto a heterologous scaffold that could preserve its helical conformation.

**Open questions:**

- Can the α$\bar{1}$-helix region of gp120 be stabilized in its prefusion conformation to study its binding to CD4?
- Can a CD4-BS2-stabilized trimer serve as an effective vaccine immunogen?
- Can specific drug inhibitors be rationally designed against CD4-BS2?

## 6. Quaternary Contact of Neutralizing Antibodies with the Env Trimer

**Figure 4:**
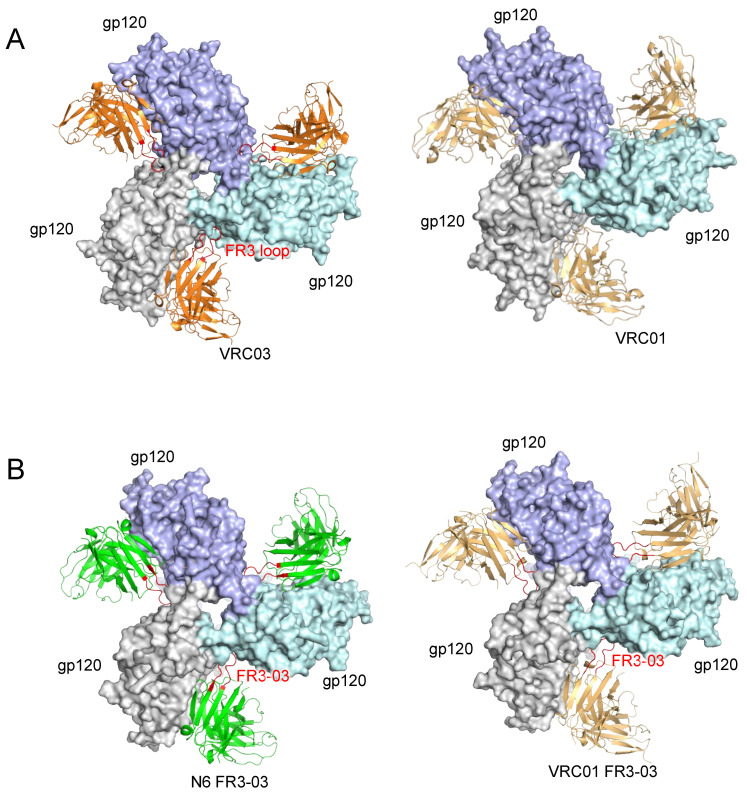
Quaternary contact of anti-CD4-BS antibodies with the Env trimer. (**A**) Structure of VRC03 (PDB ID: 6NF2) and VRC01 (PDB ID: 5FYK) complexed with SOSIP.664 trimers. The long FR3 loop of VRC03 is colored in red. (**B**) Structure of FR3-03 chimeric antibodies complexed with SOSIP.664 trimers (PDB IDs: 6NM6 and 6NNF). The engrafted FR3-03 loop is shown in red.

Selected HIV-1 neutralizing antibodies, such as PG9, PG16 and PGT145 directed against the V2-glycan supersite at the trimer apex or PGT151 directed against the gp120-gp41 interface region at the trimer base, establish quaternary contacts by interacting with two or even three different gp120 protomers of a single HIV-1 Env trimer [9,32,57,58]. While the majority of neutralizing antibodies directed to the CD4-BS bind to a single gp120 protomer, a subset of them mimics the quaternary mode of interaction of CD4 by establishing a second interaction with a neighboring gp120 protomer. In this respect, it should be emphasized that anti-CD4-BS bNAbs, like CD4, need to overcome steric restrictions from the adjacent protomer and surrounding glycans, and use very precise angles of approach in order to reach their binding epitopes in the closed Env trimer [59,60]. Two prototypic quaternary-binding anti-CD4-BS antibodies are VRC03 and VRC06, both derived from the same patient who also originated VRC01 and belonging to the same lineage [61,62]. Various mutations in CD4-BS2 impair binding of these antibodies, but the mutations with the greatest effect are charge inversions at the base of the V3 loop, which is more distally positioned in the Env spike compared to CD4-BS2 [9]. This evidence, along with the crystal structure of VRC03 bound to a SOSIP trimer [31,63], indicate that these antibodies bind to a region that overlaps with CD4-BS2 but extends toward the trimer apex to also include the V3 base (Figure 4A, left). An elongated and predominantly acidic framework 3 region (FR3) in the heavy chain of VRC03 and VRC06 was identified as the contact region responsible for interacting with the neighboring gp120 protomer. The neutralization capacity of these two antibodies is dependent on the long FR3 loop as demonstrated using deletion mutants [64]. As a comparison, VRC01 that has a regular-length FR3 is unable to reach the neighboring protomer (Figure 4A, right), and its binding to the Env trimer is not affected by mutations in CD4-BS2 [9]. Another quaternary-binding antibody with an elongated FR3 loop is 3BNC117, which contains a four-amino acid insertion in this region which allows for contact with a neighboring gp120 protomer [11]. However, FR3 elongation is not the only solution adopted by natural antibodies for establishing quaternary contact with an adjacent gp120 protomer. Two antibodies, VRC-CH31 and 1-18, interact with an adjacent gp120 protomer through an extended CDRH1 region that contains several acidic amino acids [9,33]. Conversely, antibody CH103 binds to an adjacent gp120 protomer without any extension of the FR3 region but, rather, using a unique angle of approach that orients its regular-length FR3 loop toward the second protomer. This quaternary contact has not yet been visualized by high-resolution structures, but solely predicted by aligning the monomer-bound CH103 to a soluble trimer structure and by mutagenesis experiments (Liu et al. in preparation). Interestingly, the FR3 loop of CH103 gained two acidic amino acids during the antibody lineage maturation [65]. 

The evidence that neutralizing antibodies against the CD4-BS exploit quaternary contacts to stabilize their binding inspired effort to improve the potency of HIV-1 bNAbs that lack quaternary contact by introducing such elongated loops into their FR3 region. Thus, we engrafted the elongated FR3 loop of VRC03 (FR3-03) onto several VH1-2-derived VRC01-class antibodies, namely, N6, VRC01, VRC07, VRC07-523LS and antibodies of the N49 family [64,66]. The modified antibodies showed an increased potency compared to their wild-type forms against a majority of global isolates and, in addition, exhibited an increased in vivo half-life associated with a reduced autoreactivity [64]. Structural determination of the chimeric antibody in complex with a soluble SOSIP.664 Env trimer confirmed that the engrafted FR3-03 loop establishes quaternary contact with the neighboring gp120 protomer (Figure 4B). Part of CD4-BS2, specifically K207, is involved in the quaternary contact with the FR3-03 loop while the major interface lies on the V3 base [64]. Of note, FR3-03 also contains several acidic amino acids, as seen in the CDRH1 loops of 1–18 and VRC-CH31, and in the FR3 region of CH103. The target region of all these acidic loops, whether elongated or not, is virtually the same and comprises mostly basic residues such as K207, R304, R308 and Y318. This suggests a convergent evolution of antibodies for targeting the same vulnerable epitope in the HIV-1 Env, which is also a partial mimic of the CD4 quaternary interaction with the Env trimer. We also attempted to engraft the extended CDRH1 loop of VRC-CH31 or FR3 loop from 3BNC117 to other CD4-BS bNAbs but no improvement was achieved [64]. Likewise, engrafting of the CDRH1 loop of antibody 1–18 onto VRC01-class antibodies was unsuccessful [66].

**Open questions:**

- Can the FR3-03 graft be further optimized for additional antibody improvements?
- What is the physiological basis for the reduced autoreactivity of FR3-engrafted antibodies?
- Can we design successful chimeric antibodies bearing elongated CDRH1 loops or other regions?
- Can quaternary contacts be introduced as a common strategy to improve antibodies targeting regions other than the CD4-BS?

## 7. Concluding Remarks

Formidable progress has been made over the past decade in our knowledge of the molecular anatomy of the HIV-1 Env spike and its interaction with the primary viral receptor, CD4, and potently neutralizing antibodies. Despite these advances, however, many aspects of the CD4–Env interaction and especially of the tightly regulated sequence of Env conformational changes that are triggered by such interaction remain undefined. The highly flexible and metastable conformation of the prefusion HIV-1 Env trimer has not facilitated the study of these complex structural transitions. The discovery of functionally relevant quaternary interactions of both CD4 and bNAbs with the Env trimer has opened new perspectives for elucidating the full complement of molecular events that lead to HIV-1 entry. At the same time, however, it has also opened new questions and added new complexities to an already intricate field of investigation. New study models along with further technological improvements in our investigative tools over the next few years will undoubtedly contribute to addressing these outstanding questions, which may have implications for the development of increasingly effective strategies for the ultimate control of the HIV/AIDS pandemic.

## Data Availability

Not applicable.

## References

[1] Wilen C., Tilton J.C., Doms R.W. (2012). HIV: Cell Binding and Entry. Cold Spring Harb. Perspect. Med..

[2] Chen B. (2019). Molecular Mechanism of HIV-1 Entry. Trends Microbiol..

[3] Julien J.P., Cupo A., Sok D., Stanfield R.L., Lyumkis D., Deller M.C., Klasse P.J., Burton D.R., Sanders R.W., Moore J.P. (2013). Crystal structure of a soluble cleaved HIV-1 envelope trimer. Science.

[4] Lyumkis D., Julien J.P., de Val N., Cupo A., Potter C.S., Klasse P.J., Burton D.R., Sanders R.W., Moore J.P., Carragher B. (2013). Cryo-EM structure of a fully glycosylated soluble cleaved HIV-1 envelope trimer. Science.

[5] Pancera M., Zhou T., Druz A., Georgiev I.S., Soto C., Gorman J., Huang J., Acharya P., Chuang G.Y., Ofek G. (2014). Structure and immune recognition of trimeric pre-fusion HIV-1 Env. Nature.

[6] Scharf L., Wang H., Gao H., Chen S., McDowall A.W., Bjorkman P.J. (2015). Broadly Neutralizing Antibody 8ANC195 Recognizes Closed and Open States of HIV-1 Env. Cell.

[7] Stewart-Jones G.B., Soto C., Lemmin T., Chuang G.Y., Druz A., Kong R., Thomas P.V., Wagh K., Zhou T., Behrens A.J. (2016). Trimeric HIV-1-Env Structures Define Glycan Shields from Clades A, B, and G. Cell.

[8] Gristick H.B., von Boehmer L., West A.P., Schamber M., Gazumyan A., Golijanin J., Seaman M.S., Fätkenheuer G., Klein F., Nussenzweig M.C. (2016). Natively glycosylated HIV-1 Env structure reveals new mode for antibody recognition of the CD4-binding site. Nat. Struct. Mol. Biol..

[9] Liu Q., Acharya P., Dolan M.A., Zhang P., Guzzo C., Lu J., Kwon A., Gururani D., Miao H., Bylund T. (2017). Quaternary contact in the initial interaction of CD4 with the HIV-1 envelope trimer. Nat. Struct. Mol. Biol..

[10] Chuang G.-Y., Geng H., Pancera M., Xu K., Cheng C., Acharya P., Chambers M., Druz A., Tsybovsky Y., Wanninger T.G. (2017). Structure-Based Design of a Soluble Prefusion-Closed HIV-1 Env Trimer with Reduced CD4 Affinity and Improved Immunogenicity. J. Virol..

[11] Lee J.H., Andrabi R., Su C.Y., Yasmeen A., Julien J.P., Kong L., Wu N.C., McBride R., Sok D., Pauthner M. (2017). A Broadly Neutralizing Antibody Targets the Dynamic HIV Envelope Trimer Apex via a Long, Rigidified, and Anionic beta-Hairpin Structure. Immunity.

[12] Pancera M., Lai Y.-T., Bylund T., Druz A., Narpala S., O’Dell S., Schön A., Bailer R.T., Chuang G.-Y., Geng H. (2017). Crystal structures of trimeric HIV envelope with entry inhibitors BMS-378806 and BMS-626529. Nat. Chem. Biol..

[13] Escolano A., Gristick H.B., Abernathy M.E., Merkenschlager J., Gautam R., Oliveira T.Y., Pai J., West A.P., Barnes C.O., Cohen A.A. (2019). Immunization expands B cells specific to HIV-1 V3 glycan in mice and macaques. Nature.

[14] Lee J.H., Ozorowski G., Ward A.B. (2016). Cryo-EM structure of a native, fully glycosylated, cleaved HIV-1 envelope trimer. Science.

[15] Maddon P.J., Dalgleish A.G., McDougal J., Clapham P.R., Weiss R.A., Axel R. (1986). The T4 gene encodes the AIDS virus receptor and is expressed in the immune system and the brain. Cell.

[16] McDougal J.S., Kennedy M.S., Sligh J.M., Cort S.P., Mawle A., Nicholson J.K. (1986). Binding of HTLV-III/LAV to T4+ T cells by a complex of the 110K viral protein and the T4 molecule. Science.

[17] Kowalski M., Potz J., Basiripour L., Dorfman T., Goh W.C., Terwilliger E., Dayton A., Rosen C., Haseltine W., Sodroski J. (1987). Functional regions of the envelope glycoprotein of human immunodeficiency virus type 1. Science.

[18] Lasky L.A., Nakamura G., Smith D.H., Fennie C., Shimasaki C., Patzer E., Berman P., Gregory T., Capon D.J. (1987). Delineation of a region of the human immunodeficiency virus type 1 gp120 glycoprotein critical for interaction with the CD4 receptor. Cell.

[19] Cordonnier A., Montagnier L., Emerman M. (1989). Single amino-acid changes in HIV envelope affect viral tropism and receptor binding. Nat. Cell Biol..

[20] Cordonnier A., Rivière Y., Montagnier L., Emerman M. (1989). Effects of mutations in hyperconserved regions of the extracellular glycoprotein of human immunodeficiency virus type 1 on receptor binding. J. Virol..

[21] Olshevsky U., Helseth E., Furman C., Li J., Haseltine W., Sodroski J. (1990). Identification of individual human immunodeficiency virus type 1 gp120 amino acids important for CD4 receptor binding. J. Virol..

[22] Kwong P.D., Wyatt R., Robinson J., Sweet R.W., Sodroski J., Hendrickson W.A. (1998). Structure of an HIV gp120 envelope glycoprotein in complex with the CD4 receptor and a neutralizing human antibody. Nat. Cell Biol..

[23] Kwong P.D., Wyatt R., Majeed S., Robinson J., Sweet R.W., Sodroski J., Hendrickson W.A. (2000). Structures of HIV-1 gp120 Envelope Glycoproteins from Laboratory-Adapted and Primary Isolates. Structure.

[24] Huang C.-C., Tang M., Zhang M.-Y., Majeed S., Montabana E., Stanfield R.L., Dimitrov D.S., Korber B., Sodroski J., Wilson I.A. (2005). Structure of a V3-Containing HIV-1 gp120 Core. Science.

[25] Diskin R., Marcovecchio P.M., Bjorkman P.J. (2010). Structure of a clade C HIV-1 gp120 bound to CD4 and CD4-induced antibody reveals anti-CD4 polyreactivity. Nat. Struct. Mol. Biol..

[26] Liu J., Bartesaghi A., Borgnia M.J., Sapiro G., Subramaniam S. (2008). Molecular architecture of native HIV-1 gp120 trimers. Nature.

[27] Sanders R.W., Derking R., Cupo A., Julien J.-P., Yasmeen A., De Val N., Kim H., Blattner C., De La Peña A.T., Korzun J. (2013). A Next-Generation Cleaved, Soluble HIV-1 Env Trimer, BG505 SOSIP.664 gp140, Expresses Multiple Epitopes for Broadly Neutralizing but Not Non-Neutralizing Antibodies. PLoS Pathog..

[28] Lee J.H., de Val N., Lyumkis D., Ward A.B. (2015). Model Building and Refinement of a Natively Glycosylated HIV-1 Env Protein by High-Resolution Cryoelectron Microscopy. Structure.

[29] Kong R., Xu K., Zhou T., Acharya P., Lemmin T., Liu K., Ozorowski G., Soto C., Taft J.D., Bailer R.T. (2016). Fusion peptide of HIV-1 as a site of vulnerability to neutralizing antibody. Science.

[30] Barnes C.O., Gristick H.B., Freund N.T., Escolano A., Lyubimov A.Y., Hartweger H., West A.P., Cohen A.E., Nussenzweig M.C., Bjorkman P.J. (2018). Structural characterization of a highly-potent V3-glycan broadly neutralizing antibody bound to natively-glycosylated HIV-1 envelope. Nat. Commun..

[31] Xu K., Acharya P., Kong R., Cheng C., Chuang G.-Y., Liu K., Louder M.K., O’Dell S., Rawi R., Sastry M. (2018). Epitope-based vaccine design yields fusion peptide-directed antibodies that neutralize diverse strains of HIV-1. Nat. Med..

[32] Pan J., Peng H., Chen B., Harrison S.C. (2020). Cryo-EM Structure of Full-length HIV-1 Env Bound with the Fab of Antibody PG16. J. Mol. Biol..

[33] Schommers P., Grüll H., Abernathy M.E., Tran M.-K., Dingens A.S., Gristick H.B., Barnes C.O., Schoofs T., Schlotz M., Vanshylla K. (2020). Restriction of HIV-1 Escape by a Highly Broad and Potent Neutralizing Antibody. Cell.

[34] Wang H., Cohen A.A., Galimidi R.P., Gristick H.B., Jensen G.J., Bjorkman P.J. (2016). Cryo-EM structure of a CD4-bound open HIV-1 envelope trimer reveals structural rearrangements of the gp120 V1V2 loop. Proc. Natl. Acad. Sci. USA.

[35] Ozorowski G., Pallesen J., De Val N., Lyumkis D., Cottrell C.A., Torres J.L., Copps J., Stanfield R.L., Cupo A., Pugach P. (2017). Open and closed structures reveal allostery and pliability in the HIV-1 envelope spike. Nature.

[36] Wang H., Barnes C.O., Yang Z., Nussenzweig M.C., Bjorkman P.J. (2018). Partially Open HIV-1 Envelope Structures Exhibit Conformational Changes Relevant for Coreceptor Binding and Fusion. Cell Host Microbe.

[37] Yang Z., Wang H., Liu A.Z., Gristick H.B., Bjorkman P.J. (2019). Asymmetric opening of HIV-1 Env bound to CD4 and a coreceptor-mimicking antibody. Nat. Struct. Mol. Biol..

[38] Ozorowski G., Torres J.L., Santos-Martins D., Forli S., Ward A.B. (2020). A Strain-Specific Inhibitor of Receptor-Bound HIV-1 Targets a Pocket near the Fusion Peptide. Cell Rep..

[39] Jette C.A., Barnes C.O., Kirk S.M., Melillo B., Smith A.B., Bjorkman P.J. (2021). Cryo-EM structures of HIV-1 trimer bound to CD4-mimetics BNM-III-170 and M48U1 adopt a CD4-bound open conformation. Nat. Commun..

[40] Alsahafi N., Anand S.P., Castillo-Menendez L., Verly M.M., Medjahed H., Prévost J., Herschhorn A., Richard J., Schön A., Melillo B. (2018). SOSIP Changes Affect Human Immunodeficiency Virus Type 1 Envelope Glycoprotein Conformation and CD4 Engagement. J. Virol..

[41] Castillo-Menendez L.R., Nguyen H.T., Sodroski J. (2019). Conformational Differences between Functional Human Immunodeficiency Virus Envelope Glycoprotein Trimers and Stabilized Soluble Trimers. J. Virol..

[42] Lu M., Ma X., Castillo-Menendez L.R., Gorman J., Alsahafi N., Ermel U., Terry D.S., Chambers M., Peng D., Zhang B. (2019). Associating HIV-1 envelope glycoprotein structures with states on the virus observed by smFRET. Nature.

[43] Nguyen H.T., Alsahafi N., Finzi A., Sodroski J.G. (2019). Effects of the SOS (A501C/T605C) and DS (I201C/A433C) Disulfide Bonds on HIV-1 Membrane Envelope Glycoprotein Conformation and Function. J. Virol..

[44] Stadtmueller B.M., Bridges M.D., Dam K.-M., Lerch M.T., Huey-Tubman K.E., Hubbell W.L., Bjorkman P.J. (2018). DEER Spectroscopy Measurements Reveal Multiple Conformations of HIV-1 SOSIP Envelopes that Show Similarities with Envelopes on Native Virions. Immunity.

[45] Steichen J.M., Lin Y.C., Havenar-Daughton C., Pecetta S., Ozorowski G., Willis J.R., Toy L., Sok D., Liguori A., Kratochvil S. (2019). A generalized HIV vaccine design strategy for priming of broadly neutralizing antibody responses. Science.

[46] Garces F., Lee J.H., de Val N., de la Pena A.T., Kong L., Puchades C., Hua Y.Z., Stanfield R.L., Burton D.R., Moore J.P. (2015). Affinity Maturation of a Potent Family of HIV Antibodies Is Primarily Focused on Accommodating or Avoiding Glycans. Immunity.

[47] Finzi A., Xiang S.-H., Pacheco B., Wang L., Haight J., Kassa A., Danek B., Pancera M., Kwong P.D., Sodroski J. (2010). Topological Layers in the HIV-1 gp120 Inner Domain Regulate gp41 Interaction and CD4-Triggered Conformational Transitions. Mol. Cell.

[48] Kwon Y.D., Pancera M., Acharya P., Georgiev I.S., Crooks E.T., Gorman J., Joyce M.G., Guttman M., Ma X., Narpala S. (2015). Crystal structure, conformational fixation and entry-related interactions of mature ligand-free HIV-1 Env. Nat. Struct. Mol. Biol..

[49] Ma X., Lu M., Gorman J., Terry D.S., Hong X., Zhou Z., Zhao H., Altman R.B., Arthos J., Blanchard S.C. (2018). HIV-1 Env trimer opens through an asymmetric intermediate in which individual protomers adopt distinct conformations. eLife.

[50] Munro J.B., Gorman J., Ma X., Zhou Z., Arthos J., Burton D.R., Koff W.C., Courter J.R., Smith A.B., Kwong P.D. (2014). Conformational dynamics of single HIV-1 envelope trimers on the surface of native virions. Science.

[51] Acharya P., Luongo T., Louder M.K., McKee K., Yang Y., Kwon Y.D., Mascola J.R., Kessler P., Martin L., Kwong P.D. (2013). Structural Basis for Highly Effective HIV-1 Neutralization by CD4-Mimetic Miniproteins Revealed by 1.5 Å Cocrystal Structure of gp120 and M48U1. Structure.

[52] Van Herrewege Y., Morellato L., Descours A., Aerts L., Michiels J., Heyndrickx L., Martin L., Vanham G. (2008). CD4 mimetic miniproteins: Potent anti-HIV compounds with promising activity as microbicides. J. Antimicrob. Chemother..

[53] de Taeye S.W., Ozorowski G., Torrents de la Pena A., Guttman M., Julien J.P., van den Kerkhof T.L., Burger J.A., Pritchard L.K., Pugach P., Yasmeen A. (2015). Immunogenicity of Stabilized HIV-1 Envelope Trimers with Reduced Exposure of Non-neutralizing Epitopes. Cell.

[54] Henderson R., Lu M., Zhou Y., Mu Z., Parks R., Han Q., Hsu A.L., Carter E., Blanchard S.C., Edwards R.J. (2020). Disruption of the HIV-1 Envelope allosteric network blocks CD4-induced rearrangements. Nat. Commun..

[55] Zhang P., Gorman J., Geng H., Liu Q., Lin Y., Tsybovsky Y., Go E.P., Dey B., Andine T., Kwon A. (2018). Interdomain Stabilization Impairs CD4 Binding and Improves Immunogenicity of the HIV-1 Envelope Trimer. Cell Host Microbe.

[56] Eggink D., de Taeye S.W., Bontjer I., Klasse P.J., Langedijk J.P.M., Berkhout B., Sanders R.W. (2016). HIV-1 Escape from a Peptidic Anchor Inhibitor through Stabilization of the Envelope Glycoprotein Spike. J. Virol..

[57] Wang H., Gristick H.B., Scharf L., West A.P., Galimidi R.P., Seaman M.S., Freund N.T., Nussenzweig M.C., Bjorkman P.J. (2017). Asymmetric recognition of HIV-1 Envelope trimer by V1V2 loop-targeting antibodies. eLife.

[58] Blattner C., Lee J.H., Sliepen K., Derking R., Falkowska E., de la Peña A.T., Cupo A., Julien J.-P., van Gils M.J., Lee P.S. (2014). Structural Delineation of a Quaternary, Cleavage-Dependent Epitope at the gp41-gp120 Interface on Intact HIV-1 Env Trimers. Immunity.

[59] Zhou T., Lynch R.M., Chen L., Acharya P., Wu X., Doria-Rose N.A., Joyce M.G., Lingwood D., Soto C., Bailer R.T. (2015). Structural Repertoire of HIV-1-Neutralizing Antibodies Targeting the CD4 Supersite in 14 Donors. Cell.

[60] Cheng H., Grimm S.K., Gilman M.S., Gwom L.C., Sok D., Sundling C., Donofrio G., Hedestam G.B.K., Bonsignori M., Haynes B.F. (2018). Fine epitope signature of antibody neutralization breadth at the HIV-1 envelope CD4-binding site. JCI Insight.

[61] Wu X., Yang Z.-Y., Li Y., Hogerkorp C.-M., Schief W.R., Seaman M.S., Zhou T., Schmidt S.D., Wu L., Xu L. (2010). Rational Design of Envelope Identifies Broadly Neutralizing Human Monoclonal Antibodies to HIV-1. Science.

[62] Li Y., O’Dell S., Wilson R., Wu X., Schmidt S.D., Hogerkorp C.-M., Louder M.K., Longo N.S., Poulsen C., Guenaga J. (2012). HIV-1 Neutralizing Antibodies Display Dual Recognition of the Primary and Coreceptor Binding Sites and Preferential Binding to Fully Cleaved Envelope Glycoproteins. J. Virol..

[63] Dingens A.S., Acharya P., Haddox H.K., Rawi R., Xu K., Chuang G.-Y., Wei H., Zhang B., Mascola J.R., Carragher B. (2018). Complete functional mapping of infection- and vaccine-elicited antibodies against the fusion peptide of HIV. PLoS Pathog..

[64] Liu Q., Lai Y.-T., Zhang P., Louder M.K., Pegu A., Rawi R., Asokan M., Chen X., Shen C.-H., Chuang G.-Y. (2019). Improvement of antibody functionality by structure-guided paratope engraftment. Nat. Commun..

[65] Liao H.X., Program N.C.S., Lynch R.M., Zhou T., Gao F., Alam S.M., Boyd S.D., Fire A.Z., Roskin K.M., Schramm C.A. (2013). Co-evolution of a broadly neutralizing HIV-1 antibody and founder virus. Nature.

[66] Liu Q., Zhang P., Miao H., Lin Y., Kwon Y.D., Kwong P.D., Rikhtegaran-Tehrani Z., Seaman M.S., DeVico A.L., Sajadi M.M. (2021). Rational Engraftment of Quaternary-Interactive Acidic Loops for Anti-HIV-1 Antibody Improvement. J. Virol..

